# A Comparison of Regular Consumption of Fresh Lean Pork, Beef and Chicken on Body Composition: A Randomized Cross-Over Trial

**DOI:** 10.3390/nu6020682

**Published:** 2014-02-14

**Authors:** Karen J. Murphy, Barbara Parker, Kathryn A. Dyer, Courtney R. Davis, Alison M. Coates, Jonathan D. Buckley, Peter R. C. Howe

**Affiliations:** 1 Nutritional Physiology Research Centre, University of South Australia, GPO Box 2471 Adelaide, South Australia 5001, Australia; E-Mails: kate.dyer@unisa.edu.au (K.A.D.); courtney.davis@mymail.unisa.edu.au (C.R.D.); alison.coates@unisa.edu.au (A.M.C.); jon.buckley@unisa.edu.au (J.D.B.); 2 School of Nursing and Midwifery, University of South Australia, GPO Box 2471 Adelaide, South Australia 5001, Australia; E-Mail: barbara.parker@unisa.edu.au; 3 Clinical Nutrition Research Centre, University of Newcastle, Callaghan, NSW 2308, Australia; E-Mail: peter.howe@newcastle.edu.au

**Keywords:** pork, beef, chicken, body composition, energy intake, DEXA

## Abstract

Pork is the most widely eaten meat in the world and recent evidence shows that diets high in pork protein, with and without energy restriction, may have favourable effects on body composition. However, it is unclear whether these effects on body composition are specific to pork or whether consumption of other high protein meat diets may have the same benefit. Therefore we aimed to compare regular consumption of pork, beef and chicken on indices of adiposity. In a nine month randomised open-labelled cross-over intervention trial, 49 overweight or obese adults were randomly assigned to consume up to 1 kg/week of pork, chicken or beef, in an otherwise unrestricted diet for three months, followed by two further three month periods consuming each of the alternative meat options. BMI and waist/hip circumference were measured and body composition was determined using dual energy x-ray absorptiometry. Dietary intake was assessed using three day weighed food diaries. Energy expenditure was estimated from activity diaries. There was no difference in BMI or any other marker of adiposity between consumption of pork, beef and chicken diets. Similarly there were no differences in energy or nutrient intakes between diets. After three months, regular consumption of lean pork meat as compared to that of beef and chicken results in similar changes in markers of adiposity of overweight and obese Australian middle-aged men and women.

## 1. Introduction

The global prevalence of obesity is increasing [1,2,3]. In Australia 63% of the population is overweight or obese [4]; this poses a major health concern as obesity clusters with other cardiovascular (CV) risk factors including type 2 diabetes, hypertension, hypercholesterolemia, poor mental health and physical disability, increasing risk of mortality [5]. Key strategies employed to reduce weight involve lifestyle intervention including caloric restriction and regular exercise. Particular dietary strategies shown to be effective for weight loss include energy restricted high protein diets [6,7,8,9] and using lean beef as the major protein source [8]. Until recently there has been an almost complete absence of research examining the consumption of pork and potential health benefits. This is surprising given it is the most widely eaten meat in the world [10]. In the past meat generally was perceived as a high fat food and consequently was subject to concerns regarding its potential adverse impact on health [11,12], whereas lean meat is actually low in fat and an important source of protein, iron, vitamins and minerals. There is little evidence to explain why pork consumption is low in Australia; Australians mainly consume beef and chicken. Pork is a good source of protein and recent evidence has shown that lean pork may provide CV and metabolic health benefits [13,14].

A study by Wycherley and colleagues [14], compared three diets (1) an energy restricted high pork protein diet combined with resistance exercise training; (2) a standard carbohydrate diet (control) with and without exercise; and (3) a diet matched for protein without exercise, on weight loss and body composition over a 16 week period. The authors showed that the high pork protein diet achieved the greatest losses of weight (−13.8 kg) and fat mass (−11.1 kg) and reduction in waist circumference (−13.7 cm) compared with the other two diets. There were also improvements in CV risk factors such as blood pressure, lipids, insulin and glucose with no difference between groups. We have previously shown in a pilot study that regular *ad libitum* consumption of lean pork for six months without energy restriction led to improvements in body composition compared with a habitual diet for six months (weight (pork diet: −0.8 ± 0.3 kg, habitual diet: 0.2 ± 0.5 kg), fat mass (pork diet: −0.5 ± 0.2 kg, habitual diet: 0.4 ± 0.3 kg), waist circumference (pork diet: −0.6 ± 0.4 cm, habitual diet: 0.8 ± 0.4 cm), abdominal fat (pork diet: −69 ± 24 g, habitual diet: 22 ± 26 g), %body fat (pork diet: −0.4% ± 0.2%, habitual: 0.2% ± 0.2%)) [13]. These improvements were evident after only three months of eating pork (compared with habitual diets) and were achieved without restricting energy intake. Over time dietary intake of total energy, fat, saturated fat, carbohydrate and protein decreased in both the pork and control groups but this was not significantly different. Despite these reductions in dietary intake in both groups, there were only improvements in body composition in the pork group. However, it was not possible to determine if the changes in body composition were specific to pork consumption or whether regular consumption of other high protein meats may have had the same benefit. There appears to be little difference between the nutrient (including amino acid) profile of pork and other commonly studied meats (e.g., beef, chicken *etc.*) [15], hence we sought to compare the effect of regular consumption of lean pork with that of two other commonly consumed meats in the Australian diet, namely chicken and beef, on indices of adiposity. Given there is little nutritional compositional difference between pork, beef and chicken, we did not expect any difference in body composition between the three meat groups. 

## 2. Subjects and Methods

### 2.1. Participants

Free-living overweight/obese, non-smoking men and women were recruited through local media advertisements to participate in a nine month, randomised, cross-over trial. Participants were excluded if they reported one of the following: diagnosed diabetes or CV disease; history of myocardial infarction or stroke; peripheral vascular disease; blood pressure >160/100 mmHg; liver or renal disease; anti-inflammatory, hypothyroidism, antihypertensive or hypocholesterolemic drug therapy that was not stable in the previous three months; eating >100 g fresh pork per week; inability to consume pork as required. The first eligible participant was allocated at random to one of the three meats. Subsequently, eligible participants were stratified according to gender, BMI and age by the process of minimization [16]. This study was conducted according to the guidelines laid down in the Declaration of Helsinki and all procedures involving human participants were approved by the Human Research Ethics Committee (22 June 2010) at the University of South Australia, Adelaide, Australia. Written informed consent was obtained from all participants. The trial was registered on the Australia New Zealand Clinical Trials Register (ACTRN12610000612011, 28 July 2010).

### 2.2. Study Design

**Figure 1:**
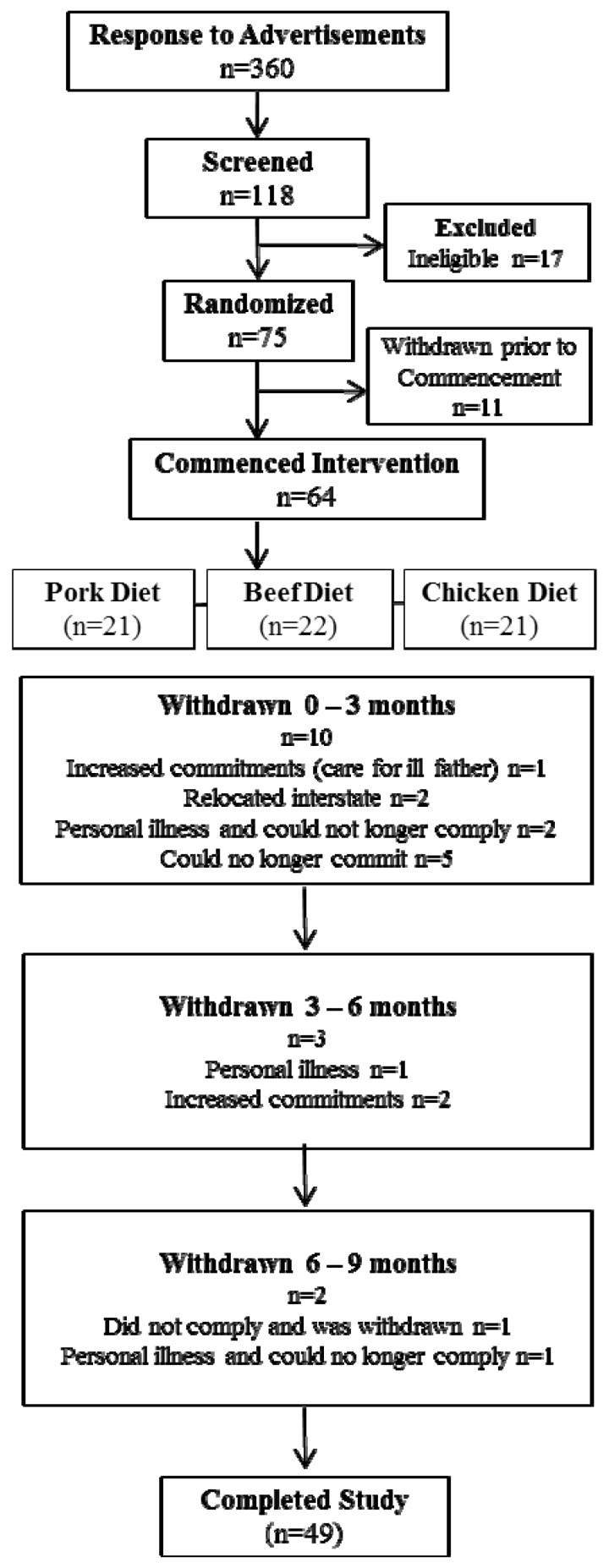
Consort diagram.

Of a total of 118 participants who were screened for eligibility, 75 were randomised to commence the intervention in the pork, beef or chicken group for the initial three months (Figure 1). At the end of this period participants crossed over to another meat for three months and then to the remaining meat for the final three month period; thus each volunteer acted as their own control. Participants attended the Nutritional Physiology Research Centre clinical trials facility at baseline and after three, six and nine months of intervention. The following assessments were made at each time point unless stated otherwise; body mass and height were recorded to calculate body mass index (BMI; kg/m^2^), waist and hip circumferences were measured using accredited protocols, body composition was assessed using dual energy x-ray absorptiometry (DEXA) and dietary intake and physical activity levels were measured.

### 2.3. Dietary Intervention

All participants were provided with five serves (women) or seven serves (men) per week of their allocated meat and asked to incorporate it into their habitual diet for each three month period. As the meats were matched on energy per serving, the portion sizes varied slightly (pork 140 g/serve, chicken 150 g/serve, beef (red meat) 150 g/serve). All participants were seen fortnightly to monitor body weight, discuss any issues arising in the intervention and collect a selection of frozen meat products including lean beef or pork steak or chicken breast, stir fry, diced and mince. All participants kept a weekly log of study meat consumption.

### 2.4. Dietary Intake

Participants were asked to record their dietary intake (consecutive two weekdays/one weekend day) in a semi-quantitative 3-day weighed food record at baseline and then at the mid-point of each three month period. Participants were asked to weigh and measure their food using scales provided. Dietary composition was analysed using a computerised database (Foodworks Professional Edition, 2009 using food composition data from AUSNUT 2007 and NUTTAB 2006; Xyris Software, Highgate Hill, Australia) where updated nutrient profiles of the study pork, beef and chicken were added to generate values for energy, macro and micronutrient consumption. The energy, moisture, ash and macronutrient composition of the meats were initially analysed by the National Measurement Institute (Victoria, Australia) to match energy composition to determine portion sizes of each type of meat. Throughout the intervention fat content was monitored from batch to batch in our laboratory using a modification of the Bligh and Dyer [17] method. An average of values determined from different batches was used to determine the study meat profile. Frequency of consumption of energy and macro and micronutrients were estimated using a 74-item food frequency questionnaire (FFQ) [18] at baseline and at the end of each meat phase. The FFQ requests information relating to food choices, portion size, quantity and consumption frequency of different food and beverage items and is a validated and reliable measure of dietary intake for use in epidemiological studies within the Australian population [19,20,21]. Compliance was assessed by comparison of daily meat consumption logs and 3-day weighed food records and FFQ collected at baseline and at the end of each meat phase.

### 2.5. Physical Activity

Participants recorded a diary of all physical activity conducted in a 24 h period over three days (two weekdays and one weekend day) [22]. Energy expenditure (kcal) was then calculated for every 15 min period in a 24 h day according to nine categories of different types of activity (e.g., sleeping, playing sports, gardening *etc.*) and multiplied by the appropriate physical activity level factor for the reported intensity of exercise. This was multiplied by body weight and then averaged for three days [22]. 

### 2.6. Body Mass Index and Body Composition

Each participant’s height and mass were recorded to calculate BMI (kg/m^2^). Height was measured to the nearest 0.1 cm whilst barefoot using a wall-mounted stadiometer (SECA; Vogel and Halke, Hamburg, Germany). Body mass was measured to the nearest 0.1 kg with participants wearing light clothing using the TANITA Ultimate Scale 2000 (Tanita Corporation, Tokyo, Japan).Waist and hip measurements were taken using a metric tape measure according ISAK international guidelines, as described by Norton and Olds [23] and the waist to hip ratio (WHR) was calculated. Participants had their percentage of body fat, fat mass (kg), abdominal fat (g) and lean mass (% and kg) assessed using DEXA (Lunar Prodigy, General Electric, Madison, WI, USA). Abdominal fat content was generated using Lunar Prodigy software from regional analysis of the DEXA scan to assess the region from the top of the iliac crest, with the lateral borders extending to the edge of the abdominal soft tissue, and the upper margin 20% above the pelvis between the pelvis and the neck borders.

### 2.7. Statistical Analysis

Based on previous determinations of the variance in the primary outcome measure (change of % body fat from baseline to three months), we estimated that a total of 51 participants would give 80% power to detect a significant (*p* < 0.05) 1% difference in change in percentage body fat between dietary treatments or a 2 kg change in body weight at an alpha level of 0.05. Data of participants who completed the trial were checked for normality and then analysed using Random-effects GLS Regression to identify differences between means where significant main effects were seen. Analysis focused on changes in indices of adiposity at the end of each dietary phase using STATA Statistics Data analysis 11 (StataCorp, Texas, USA). Data are presented as means ± SEM (standard error of mean). To allow for multiple comparisons, significance was set at *p* < 0.003 for dietary intake data and *p* < 0.006 for anthropometry and body composition data.

## 3. Results

### 3.1. Participant Characteristics

75 participants were enrolled from the 101 participants deemed eligible, to allow for an approximate 40% withdrawal rate. Of the 75 participants who were enrolled in the intervention, 11 withdrew prior to commencement (due to change of mind and increased personal commitments) and 15 withdrew after commencement (Figure 1). Reasons for withdrawal included inability to commit to the study (*n* = 5), personal illness (*n* = 4), relocated interstate (*n* = 2) increased time commitments (*n* = 3) and one participant was withdrawn due to lack of compliance with the protocol. Thus 49 participants completed the full 9-month intervention period with characteristics presented in Table 1. This population were on average middle aged (50 ± 2 years), obese (BMI 30.5 ± 0.5 kg/m^2^) with waist circumferences (WC) above the recommended cut off point (103 ± 11 cm) [5,24]. 49% of the population was obese and 51% overweight.

**Table 1:** Gender, age, anthropometric measurements, body composition, daily dietary intake and energy expenditure of study population at baseline.

	Mean ± SD
Gender *n*	24 M/25 W
Age (years)	50 ± 2
Height (m)	1.72 ± 0.1
Weight (kg)	90 ± 14
BMI (kg/m^2^)	30.5 ± 3.6
WC (cm)	102.6 ± 11.3
	108.5 ± 8.2 M/96.9 ± 11.0 W
HC (cm)	110.3 ± 10.1
	106.7 ± 5.4 M/113.7 ± 12.3 W
WHR	0.93 ± 0.1
	1.02 ± 0.07 M/0.86 ± 0.07 W
% Body Fat	49.4 ± 6.3
Fat mass (kg)	35.3 ± 8.5
Abdominal fat (g)	3655 ± 1075
Lean mass (kg)	50.1 ± 9.8
*Energy Expenditure*	
EExp (MJ)	16.3 ± 3.2
EExp (kcal)	3889 ± 753
*Dietary Intake*	
Energy (MJ)	9.3 ± 3.0
Energy (kcal)	2222 ± 691
Protein (g)	103 ± 29
%en Protein	19 ± 3.4
CHO (g)	227 ± 70
%en CHO	41 ± 6.2
Fat (g)	89 ± 38
%en Fat	34 ± 6.4
SFA (g)	34 ± 13
%en SFA	14 ± 3.1
MUFA (g)	34 ± 17
PUFA (g)	14 ± 10
Alcohol (g)	10 ± 13
%en Alcohol	3 ± 4
Iron (mg)	13 ± 4
Zinc (mg)	14 ± 7

Dietary intake was captured using 3-day weighed food records and energy expenditure was estimated using three day physical activity diaries.

### 3.2. Pork, Beef and Chicken Consumption

**Figure 2:**
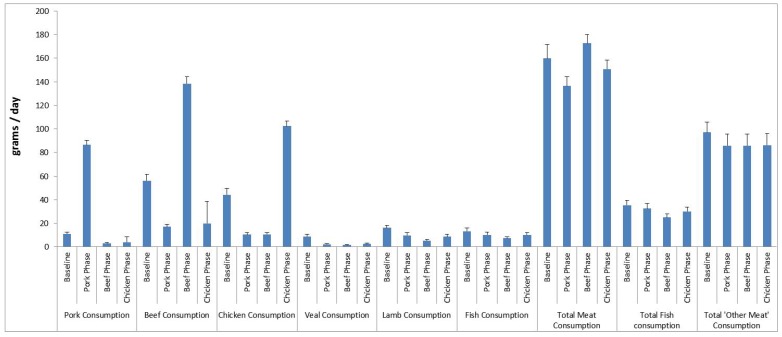
Average meat and fish consumption (grams per day ± SEM) from the Cancer Council of Victoria Food Frequency Questionnaire at baseline and for each dietary phase (beef, pork, chicken) *n* = 49.

According to the FFQ, average daily consumption of pork, beef and chicken in the relevant phase was 87 g (609 g/week), 138 g (966 g/week) and 102 g (714 g/week), respectively. Total meat consumption, total fish consumption and consumption of lamb and veal did not change during the intervention (Figure 2). Total meat consumption (sum of pork, chicken, beef, veal and fish) was 137 g/day for the pork group, 173 g/day for the beef group and 151 g/day for the chicken group. The consumption of provided pork, beef and chicken was calculated from the daily meat consumption logs. The average consumption of pork, beef and chicken in the relevant phase was 119 ± 21 g/day (832 ± 146 g/week), 129 ± 23 g/day (900 ± 161 g/week) and 129 ± 26 g/day (900 ± 180 g/week), respectively. The discrepancy between the consumption of meats reported in the FFQ and the consumption logs is due to the serving sizes for pork, beef and chicken according to the Cancer Council of Australia FFQ being 78 g, 132 g, 92 g, respectively, whereas the serving sizes of pork, beef and chicken provided in the present study were 140 g, 150 g, 150 g, respectively. Based on the daily meat logs compliance to the pork, beef and chicken diets were 97%, 98% and 97%, respectively.

SEM, standard error of the mean. ^a^ Total meat consumption is the sum of: pork + beef + chicken + veal + lamb + bacon + ham + sausages + salami + fish + fried fish + tinned fish; ^b^ Total fish consumption is the sum of: fish + fried fish + tinned fish; ^c^ Total “other meat” consumption is the sum of: pizza + pasta + hamburger + meat pie.

**Table 2:** Mean values for energy and nutrient intake from weighed food records * at the end of each diet phase (Pork, Beef, Chicken), *n* = 49 and difference between meats (95% confidence intervals).

	Pork	Beef	Chicken	ΔBeef-Pork ^a^	P value	ΔChicken-Pork ^b^	*p* value
Energy (kJ)	8830 ± 373	8414 ± 383	8370 ± 392	−416 (−1119, 286)	0.245	−460 (−1162, 242)	0.199
Energy (kcal)	2111 ± 89	2011 ± 92	2001 ± 94	−100 (−267, 68)	0.245	−110 (−278, 58)	0.199
Protein (g)	103 ± 4	104 ± 4	100 ± 5	0.8 (−7.2, 8.8)	0.848	−2.9 (−10.9, 5.1)	0.475
%en Protein	20 ± 0.5	21 ± 0.5	21 ± 0.5	1.2 (0.1, 2.3)	0.036	0.5 (−0.6, 1.7)	0.343
CHO (g)	218 ± 10	207 ± 12	201 ± 10	−10.8 (−32.2, 10.7)	0.325	−16.8 (−38.3, 4.7)	0.125
%en CHO	42 ± 1	41 ± 1	40 ± 1	−0.8 (−3.1, 1.4)	0.472	−1.4 (−3.6, 0.8)	0.222
Fat (g)	77 ± 5	71 ± 5	75 ± 5	−5.7 (−14.6, 3.2)	0.207	−1.2 (−10.1, 7.7)	0.789
%en Fat	31 ± 1	30 ± 1	33 ± 1	−0.8 (−2.8, 1.3)	0.458	1.5 (−0.6, 3.5)	0.157
SFA (g)	30 ± 2	27 ± 2	29 ± 2	−3.5 (−8.0, 1.0)	0.128	−0.9 (−5.4, 3.6)	0.689
%en SFA	12 ± 0.6	11 ± 0.5	13 ± 0.5	−0.9 (−2.1, 0.2)	0.104	0.3 (−0.8, 1.5)	0.573
MUFA (g)	30 ± 2	27 ± 2	29 ± 2	−2.3 (−6.3, 1.8)	0.272	−1.0 (−5.1, 3.0)	0.615
PUFA (g)	11 ± 1	12 ± 1	12 ± 1	0.3 (−1.3, 1.8)	0.714	1.2 (−0.4, 2.7)	0.143
Alcohol (g)	14 ± 3	12 ± 2	10 ± 3	−1.7 (−5.4, 2.0)	0.366	−3.2 (−6.9, 0.5)	0.089
%en Alcohol	4.3 ± 0.8	4.5 ± 0.9	3.5 ± 0.8	0.2 (−0.9, 1.2)	0.732	−0.8 (−1.8, 0.3)	0.139
Iron (mg)	12.3 ± 0.5	14.0 ± 0.7	11.9 ± 0.6	1.7 (0.5, 2.9)	0.005	−0.4 (−1.6, 0.8)	0.533
Zinc (mg)	11.6 ± 0.5	15.7 ± 0.8	11.5 ± 0.8	4.1 (2.4, 5.7)	*p* < 0.0001	−0.2 (−1.8, 1.5)	0.844
EExp (MJ)	16.5 ± 0.5	16.2 ± 0.5	16.3 ± 0.5	−0.268 (−0.76, 0.22)	0.284	−0.086 (−0.58, 0.41)	0.734
EExp (kcal)	3933 ± 116	3870 ± 112	3903 ± 119	−64 (−182, 53)	0.284	−21 (−139, 98)	0.734

* Mean ± standard error of the mean. Abbreviations: kJ, kilojoule; kcal, kilocalorie %en, percent energy; SFA, saturated fatty acid; MUFA, monounsaturated fatty acid; PUFA, polyunsaturated fatty acid; g, grams; mg, milligrams; µg, micrograms; EExp, energy expenditure; MJ, megajoule; ^a^ Difference between beef and pork adjusting for chicken (95% Confidence Intervals) according to random-effects GLS regression; ^b^ Difference between chicken and pork adjusting for beef (95% Confidence Intervals) according to random-effects GLS regression. *p* < 0.003 was considered significant to allow for multiple comparisons. No significant differences were reported for any variable.

**Table 3:** Mean values for anthropometric measurements and body composition * at the end of each diet phase (Pork, Beef, Chicken), *n* = 49 and difference between meats (95% confidence intervals).

	Pork	Beef	Chicken	Difference betweenPork and Beef ^a^	*p* value	Difference betweenPork and Chicken ^b^	*p* value
Weight (kg)	89 ± 2	89 ± 2	89 ± 2.0	−0.003 (−0.609, 0.602)	0.991	−0.018 (−0.624, 0.587)	0.953
BMI (kg/m^2^)	30.1 ± 0.5	30.1 ± 0.5	30.1 ± 0.5	−0.009 (−0.223,0.205)	0.934	−0.006 (−0.220, 0.208)	0.957
WC (cm)	101.0 ± 1.6	101.3 ± 1.6	101.3 ± 1.6	0.360 (−0.455, 1.18)	0.387	0.314 (−0.501, 1.13)	0.450
HC (cm)	109.8 ± 1.5	109.3 ± 1.5	109.7 ± 1.4	−0.475 (−1.064, 0.115)	0.115	−0.148 (−0.738, 0.441)	0.622
WHR	0.925 ± 0.016	0.932 ± 0.016	0.929 ± 0.016	0.007 (0.0001, 0.014)	0.046	0.004 (−0.003, 0.011)	0.222
% Body Fat	49.0 ± 0.9	48.9 ± 0.9	49.0 ± 0.9	−0.02 (−0.558, 0.518)	0.942	0.052 (−0.486, 0.590)	0.850
Fat mass (kg)	35.3 ± 1.3	35.4 ± 1.3	35.4 ± 1.3	0.098 (−0.418, 0.613)	0.710	0.057 (−0.459, 0.573)	0.828
Abdominal fat (g)	3495 ± 149	3486 ± 149	3500 ± 147	−8.68 (−82.15, 64.79)	0.817	5.47 (−68.0, 78.94)	0.884
% Lean Mass	60.4 ± 1.0	60.3 ± 1.0	60.4 ± 1.0	−0.078 (−0.482, 0.327)	0.707	−0.008 (−0.413, 0.397)	0.968
Lean mass (kg)	53.7 ± 1.5	53.6 ± 1.5	53.6 ± 1.5	−0.096 (−0.445, 0.253)	0.590	−0.07 (−0.419, 0.280)	0.696

* Mean ± standard error of the mean. Abbreviations: BMI, body mass index; WC, waist circumference; HC, hip circumference; WHR, waist/hip ratio; ^a^ Difference between pork and beef adjusting for chicken (95% Confidence Intervals) according to random-effects GLS regression; ^b^ Difference between pork and chicken adjusting for beef (95% Confidence Intervals) according to random-effects GLS regression. *p* < 0.006 was considered significant to allow for multiple comparisons. No significant differences were reported for any variable.

### 3.3. Dietary Intake and Physical Activity

Total energy and macronutrient intakes were adjusted for the nutrient profile of the provided pork, beef and chicken. There was no difference in intake of energy, macronutrients (total fat, protein or carbohydrate) or micronutrients in either group over time (Table 2) with the exception of zinc. The difference between the beef and pork group (*p* < 0.0001) most likely represents the greater zinc composition of beef (beef 3.7 mg/100 g *vs.* pork 1.7 mg/100 g, Foodworks Professional Edition). These data indicate that all participants were substituting meats in their diet without altering total energy or total protein intake. There was no difference in total energy expenditure (MJ/day) according to the physical activity diaries, indicating that participants did not change their physical activity levels and subsequent energy expenditure during the intervention (Table 2). 

### 3.4. Body Composition

There was no difference in any index of adiposity, nor was there any change in lean mass, between groups over time (Table 3). While there was a slight reduction in WC and WHR in the pork group compared to beef and chicken groups (*p* = 0.046), these were not significant when allowing for multiple comparisons.

## 4. Discussion

Previous research has focused on relationships between the consumption of lean beef and increased satiety and weight loss [25] however several of these studies have utilised hypocaloric, high protein diets specifically designed for weight loss. Until recently there has been little research published demonstrating the cardiometabolic health benefits of consuming pork. Despite pork being the most frequently consumed meat in Europe, it is consumed less frequently than other meats in some cultures such as Australia. We can only speculate the reason why pork consumption is low in Australia, such as it may be perceived as a fattier and less healthy meat choice or be associated with increased disease risk (*i.e.*, cardiovascular disease) [12]. For example a study by Lea and Worsley [26] surveyed 707 Australians (*n* = 601 omnivores, *n* = 106 vegetarians) and showed 10%–12% of non-vegetarian men and women (*n* = 540) thought red meat such as beef or lamb was fattening. While there is a lack of evidence linking pork with this perception, anecdotal evidence suggests that Australians may perceive pork as an unhealthy meat choice, which may explain why pork consumption is relatively low in Australia while beef and chicken are the major meats consumed [27]. 

We and others have recently shown that regular consumption of fresh lean pork may improve body composition [13,14]. However we were unable to say if improvements in body composition were specific to pork or whether consumption of other lean meats may have had the same benefit. The present study found no significant difference in any measure of body composition between the pork, beef or chicken diets, although pork was associated with reductions in WC and WHR compared with the other meats, but the magnitude of difference was small and did not remain statistically significant when allowing for multiple comparisons. One might argue that three months is too short a time to observe any impact of meat consumption on adiposity. In our preceding study, however, we observed more than 1 kg weight loss (which was almost entirely loss of fat) after only three months of eating pork compared with a customary diet [11]. The extremely small differences in the present study indicate that neither a larger study nor a longer intervention period would be likely to demonstrate differences in adiposity following consumption of these different meats matched for energy content. 

Our study is in agreement with results from Melanson *et al*. [28] who conducted a 12-week randomised, controlled trial where overweight women consumed an energy restricted diet with either lean beef or chicken as the major protein source together with undertaking moderate exercise. The authors reported no difference in weight loss or % body fat or blood lipid profiles following a beef or chicken diet. Similarly, Mahon and colleagues [29] compared consumption of lean beef or chicken as the primary protein source in a hypocaloric diet in 61 obese females. These authors found no difference in the amount of weight loss, fat loss and reduction in low density lipoprotein cholesterol after 12 weeks consumption of either a chicken or beef diet. Similarly, a cholesterol-lowering study by Davidson *et al.* [30] compared a NCEP Step 1 diet (National Cholesterol Education Program Step 1 diet) containing 170 g of lean meat (pork, veal and beef) with a diet containing lean poultry and fish as the primary meat for 36 weeks. The authors showed no difference in the change in serum lipid levels between groups. Moreover Coates *et al.* [31] showed that consumption of 1 kg of fresh pork per week for 12 weeks did not change body weight. Finally an acute satiety study by Charlton and colleagues [32] compared the consumption of pork, beef and chicken on acute satiety and appetite regulatory hormones and showed no difference between meats.

There are conflicting perceptions of health impacts of pork consumption and, while there does not appear to be any published evidence showing an increased risk of obesity or CV disease, levels of pork consumption in Australia are still lower than other meats (72 g/day) [27]. Consumption of beef and chicken is more widely accepted; these appear to be the two most commonly consumed meats in the Australian diet (93 g/day and 99 g/day, respectively) [33]. Perhaps it is because lean beef has been shown to help with weight loss and lean chicken breast is a regular component of weight loss diets [7,8]. However there is constant discussion about the association between meat consumption and development of coronary heart disease (CHD), most likely due to concern over the saturated fat content. Recently Micha and colleagues [34] published a systematic review and meta-analysis of the evidence for relationships between unprocessed fresh meat from beef, hamburgers, lamb, pork or game and processed meat (salami, sausages, hot dogs, bacon or processed luncheon meats) and fresh (red) meat and CHD. They found that the intake of unprocessed (red) meat was not associated with CHD (*n* = 56,311 participants *n* = 769 events, relative risk = 1.00/daily serving, 95% CI, 0.81 to 1.23 with no statistically significant heterogeneity between studies *p* = 0.36), whereas each daily serving of processed meat was associated with 42% higher risk of CHD (*n* = 614,062 participants *n* = 21,308 events, relative risk = 1.42, 95% CI, 1.07 to 1.89 with statistically significant heterogeneity between studies *p* = 0.04). While some evidence is present of a relationship between consumption of total meat and increased CHD risk [34] the magnitude and effect is largely dependent on the type of meat and study outcome. Taken together, there is a need for greater understanding of the potential health benefits of fresh lean meat. Recognition of potential health benefits in dietary recommendations especially from a consumer perspective is vital as consumer’s attitudes towards pork consumption is likely to be influenced by the link between food and health [35].

## 5. Conclusions

We acknowledge limitations of this study, firstly of cross-over design and no implementation of a washout period. This design did not include a wash-out period as it was deemed that three months on each phase was sufficient for volunteers to reach steady state prior to the assessment conducted at the end of the three month meat phase. Secondly, there are well known limitations associated with collecting accurate dietary data hence why we collected FFQ data together with 3-day WFR and daily meat consumption logs to monitor compliance and calculate energy and nutrient intake. 

The current study provides further evidence that three months of regular consumption of lean pork as compared to that of lean beef and chicken results in similar changes in body composition. Thus the perception that pork is an inferior meat in terms of nutrition should be reconsidered.

## Abbreviations

BMI: body mass index
CHD: coronary heart disease
CV: cardiovascular
DEXA: dual energy xray absorptiometry
FFQ: food frequency questionnaire
SEM: standard error of mean
SD: standard deviation
WC: waist circumference
WHR: waist to hip ratio
